# Association between imaging parameter changes and triangular fibrocartilage complex injury after distal radius fractures

**DOI:** 10.1186/s13018-023-04438-5

**Published:** 2023-12-09

**Authors:** Chunye Tan, Zeyu Wang, Linwei Li

**Affiliations:** 1 First People’s Hospital of Changzhou Affiliated to Soochow University, Changzhou, Jiangsu People’s Republic of China; 2 Department of Trauma Center and Orthopedic Surgery, The First People’s Hospital of Changzhou Affiliated to Soochow University, Juqian Road 185, Changzhou, 213000 Jiangsu People’s Republic of China

**Keywords:** Triangular fibrocartilage complex (TFCC) injury, Radiologic parameter, Distal radius fracture, Radius length, DRUJ distance

## Abstract

**Background:**

Triangular fibrocartilage complex (TFCC) injury is a frequent soft tissue injury that has been observed to accompany distal radius fractures (DRFs) with concomitant changes in radiologic parameters. The aim of this study was to investigate the relevance of distal radial radiologic parameters associated with DRF and traumatic TFCC injury.

**Methods:**

A total of 172 patients with distal radius fractures who underwent X-ray, CT, and MRI before undergoing volar locking plate or external splint fixation between October 2021 and December 2022 were included in this study. An analysis of various radiologic parameters and the classification of fracture type and TFCC injuries by CT and MRI was performed. All patients were divided into the TFCC uninjured group and the injured group. The incidence and relevant radiologic parameters were compared.

**Results:**

This study included 76 males and 96 females with a mean age of 56.1 years. Among all patients, 33 (19.2%), 40 (23.2%), and 99 (57.6%) had DRF with A, B, and C fractures, respectively, according to the AO/OTA classification. In patients with fractures, the TFCC was found to be injured in 54.1% (93/172) of patients (type 1A in 21, 1B in 46, 1C in 39, and 1D in 35) but uninjured in 45.9% (79/172). There were significant differences between the TFCC injured and uninjured groups regarding the radius length (*p* = 0.044) and DRUJ distance (*p* = 0.040) of radiologic parameters that changed with DRF, although there were no differences between the two groups regarding gender, age, injured side, intra- and extra-articular, radius inclination and palmer tilt angle, or sagittal translation. Within the TFCC injured group, the radius length and DRUJ distance were 4.83 mm and 2.95 mm less or wider than 7.19 mm and 1.83 mm of the uninjured group. Moreover, shorter radius length was related to type lB TFCC injury (*p* = 0.041). Both radius length (AUC = 0.658) and DRUJ distance (AUC = 0.582) had no convincing predictive value for TFCC injury in DRF.

**Conclusion:**

1B TFCC injury is most common in patients with DRF and concomitant TFCC injury. Both radius length and DRUJ distance have a significant statistical correlation with TFCC injury, and patients with TFCC injury tend to have a shortened radius and wider DRUJ distance, although they have no predictive value for TFCC injury in DRF. In addition, a shorter radius length was related to type lB TFCC injury.

## Introduction

Distal radius fracture (DRF) is one of the most common fractures, particularly as the population ages and its incidence rises year after year in many nations and regions [1, 2]. The instability of the distal ulnar radial joint (DRUJ) is still present in up to 37% of patients, although the majority of patients have had their wrist function significantly recovered through conservative or surgical treatment [3, 4].

The triangular fibrocartilage complex (TFCC) is one of the most important stabilizing structures of the DRUJ [5]. Reconstruction of deep fibers of the TFCC is beneficial for stabilizing the wrist joint [6]. During clinical practice, it is relatively easy for physicians to see distal ulnar radius fractures on wrist X-rays or CT. Nevertheless, when we examine patient care or if the patient has an MRI, we can find that some patients have concomitant traumatic TFCC injury. Studies have shown that 49–78% of patients with DRF also have a combined traumatic TFCC injury [7–9]. This has a significant impact on the stability of the patient’s DRUJ postoperatively and the development of chronic, recurrent pain in the wrist joint and postoperative joint stiffness. Even so, the insidious nature of TFCC injury, which is still very easy to overlook in clinical work, results in underdiagnosis.

There appears to be a correlation between changes in bony structures in DRF and TFCC injury. It has been shown that radial length loss of more than 5–7 mm can cause excessive tension in the inferior ulnar radial ligament on the palmar and dorsal sides resulting in ligamentous tear injury [10]. A cadaveric study has shown that DRF with a dorsal angle of more than 20° tends to cause injury at the structural attachment of the TFCC and affect the stability of the inferior ulnar radial joint [11]. In addition, biomechanical studies point out that distal radius palmar angulation greater than 10° and dorsal angulation greater than 10–20° can cause TFCC injury [12]. However, because no data on the clinical outcome were utilized in those studies, they are lacking from a clinical perspective.

The objective of this study was to investigate the relevance of distal radial radiological parameters associated with DRF and traumatic TFCC injury through a retrospective study and to illuminate whether the location of the distal radius in patients with fractures can predict the presence of traumatic TFCC injury, which is helpful to further understand of patients’ conditions as well as clinical work.

## Materials and methods

Retrospective research was used in this study. All patients were admitted to the emergency department of the First People’s Hospital of Changzhou from October 2021 to December 2022. The inclusion criteria were as follows: age > 18 years, a confirmed DRF (AO classification), unilateral fresh fracture (< 14 days after fracture occurrence), emergency X-ray without manual reduction, no previous surgical history of the injured wrist, and no accompanying fracture of the injured limb. The exclusion criteria were old fracture, systemic bone disease (e.g., hyperparathyroidism) or local disease (e.g., tumor, Paget’s disease, or rheumatoid arthritis), treatment other than VLP or EF, loss to follow-up or incomplete data. A total of 172 patients with complete imaging data, including preoperative X-ray, CT, and MRI, were collected. All patients underwent surgical treatment after a complete examination.

**Fig. 1:**
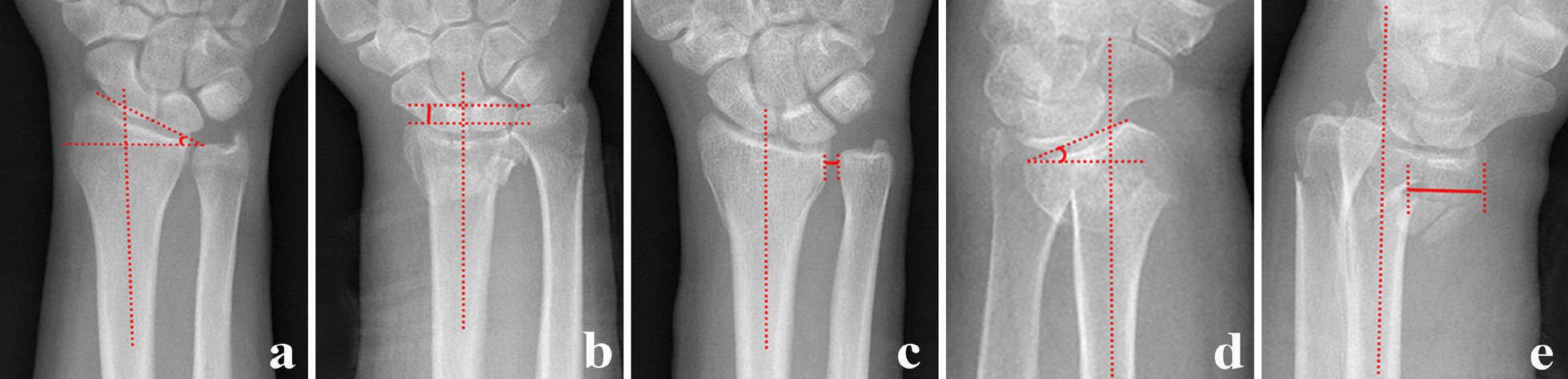
Radiologic parameter measurement of wrist joints. **a** Radius inclination. **b** Radius length. **c** DRUJ distance. **d** Palmer tilt angle. **e** Sagittal translation. DRUJ, distal radioulnar joint

The radiologic parameters based on AP and lateral X-rays of bilateral wrist joints (injured and uninjured) mainly include radial length (RL), radius inclination, DRUJ distance, palmar tilt angle, and sagittal translation of the distal radius (Fig. 1). All measurements were repeated by three orthopedic surgeons to ensure their reliability. The radial length was calculated as the distance between two lines made on the AP projection from the level of the ulnar aspect of the articular surface and the apex of the radial styloid, perpendicular to the long axis of the radius [13]. The angle between the vertical line of the long axis of the radius, the middle of the ulnar sigmoid notch of the radius, and the top of the radial styloid process was referred to as the radius inclination. The DRUJ distance was defined as the greatest distance between the volar or dorsal cortical rim of the sigmoid notch of the radius and the ulnar head. The palmar tilt angle was the angle formed by the vertical line of the long axis of the radius and the line joining the most distant point of the distal radial articular surface on the palmar and dorsal sides. Therefore, on the lateral X-ray, we defined sagittal translation as the distance between the volar cortex of the radius shaft and the volar cortical margin of the distal fracture fragment [13, 14]. X-rays of the uninjured wrist helped us better understand the normal anatomic angle of the radius.

The type of DRF was classified based on radiographs and CT imaging by orthopedic surgeons in accordance with the AO/OTA classification [15]. As part of regular clinical scans for pathologies unrelated to the radius itself, all images were obtained using a CT scanner (Philips, Colorado, USA) with excellent clarity. These CT images had a 0.5 mm slice spacing. Three-dimensional (3-D) modeling software (SESANS RIS, ver. 2.1.1; Changzhou, China) was used to take each wrist image from the Picture Archiving and Communication System, anonymize it, and segment it.

According to the normal MR procedure for imaging the wrist in our facility, all patients underwent MRI of the wrist in the coronal, sagittal, and axial planes on an Ingenia 3.0 T machine (Philips, Colorado, USA). All patterns had a 10% interslice gap and a 2 mm slice width. Orthopedic surgeons still read and analyzed the picture by SESANS software. Palmer’s classification was used to evaluate traumatic TFCC injury [16]. To summarize, TFCC 1A injuries were defined as perforations of the articular disk, TFCC 1B injuries as tears of the ulnar side of the TFCC and detachment from the ulnar fovea, TFCC 1C injuries as distal tears in the palmar side of the TFCC, and TFCC 1D injuries as radial tears that tore the origin of the TFCC directly off the sigmoid notch.

For statistical analyses, age, radiologic parameters, and other continuous factors were represented as the mean and standard variation for statistical analyses (SD). The difference was assessed using either Student’s t test or ANOVA, as necessary. The categorical factors, such as the gender distribution, were represented as numbers and percentages, and the Pearson chi-square test was used, as necessary, to assess the difference between the two groups. The threshold of the statistical test was fixed at 0.05. All studies were carried out using SPSS 21.0 software (IBM, NY, USA). Consequently, ROC curve analysis was carried out using version 20.218 of the MedCalc Statistical Program (MedCalc Software Ltd, Mariakerke, Belgium).

## Results

A total of 172 patients were enrolled in this study. Detailed patient fundamental data are shown in Table 1. This study comprised a total of 76 men and 96 women, with a mean age of 56.1 years. There were 33 extra-articular fractures (A2 (*n* = 9), A3 (*n* = 24)), 40 partial articular fractures (B1 (*n* = 15), B2 (*n* = 4), B3 (*n* = 21)), and 99 complex articular fractures (C1 (*n* = 29), C2 (*n* = 29), C3 (*n* = 41)) according to the data from the AO/OTA classification. Therefore, according to Palmer’s classification, 21 patients had 1A traumatic TFCC injury, 46 patients had 1B traumatic TFCC injury, 39 patients had 1C traumatic TFCC injury, and 35 patients had 1D traumatic TFCC injury.

**Table 1:** Demographic data from patients with distal radius fracture and traumatic TFCC injury

Total	172 patients
Sex (male/female)	76:96
Age	56.1
Injured side (L:R)	83:89
Fracture type (intra/extra-articular)	139:33
*AO classification*	
A2	9 (5.2%)
A3	24 (14.0%)
B1	15 (8.7%)
B2	4 (2.3%)
B3	21 (12.2%)
C1	29 (16.9%)
C2	29 (16.9%)
C3	41 (23.8%)
*Palmer’s classification*	
1A	21
1B	46
1C	39
1D	35

*L* Left; *R* Right; *AO* Arbeitsgemeinschaft fur osteosynthesefragen

Variable types of distal radius fractures have variable rates of traumatic TFCC injury. In the 172 cases shown in Table 2, the general incidence was 54.1%. In patients with complete intra-articular fractures of type C, the rate of TFCC injury was comparatively high. The injury rate of TFCC in type C3 was 75.6%. Additionally, the TFCC injury incidence for type B3 was 61.9%. Different types of TFCC injury can coexist in the same type of fracture, such as 1B and 1D TFCC injury in type B3. Due to the relatively limited number of cases or the low occurrence of this type of TFCC injury, some fracture types, such as A2 and B2, may only comprise one or two types of TFCC injury. Overall, this study’s incidence of type 1B TFCC injury was 26.7%.

**Table 2:** Incidence and classification of TFCC injury in uninured and injured group

AO	Uninjured group	TFCC injury (Palmar classification)				Injured	Total
		1A	1B	1C	1D		
A2	7	0 (0)	2 (22.2%)	0(0)	1 (11.1%)	2	9 (22.2%)
A3	13	3 (12.5%)	7 (29.2%)	2 (8.3%)	6 (25.0%)	11	24 (45.8%)
B1	11	1 (6.7%)	4 (26.7%)	1 (6.7%)	1 (6.7%)	4	15 (26.7%)}
B2	3	1 (25.0%)	0(0)	0(0)	0(0)	1	4 (25.0%)
B3	8	4 (19.0%)	6 (28.6%)	3 (14.3%)	8 (38.1%)	13	21 (61.9%)
C1	15	3 (10.3%)	6 (20.7%)	7(24.1%)	5 (17.2%)	14	29 (48.3%)
C2	12	5 (17.2%)	8 (27.6%)	10(34.5%)	8 (27.6%)	17	29 (58.6%)
C3	10	4 (9.8%)	13(31.7%)	12 (29.3%)	6 (14.6%)	31	41 (75.6%)
Total	79	21 (12.2%)	46 (26.7%)	39 (22.7%)	35 (20.3%)	93	172 (54.1%)

*AO* Arbeitsgemeinschaft fur osteosynthesefragen; *TFCC* Triangular fibrocartilage complex

The demographic data of the two groups are shown in Table 3. The TFCC uninjured group included 79 patients, while the TFCC injured group included 93 patients. There were no significant differences between the two groups regarding demographic data, fracture type, or incidences of intra- or extra-articular fractures. However, according to radiologic parameters, there were significant differences between the two groups regarding radius length and DRUJ distance. The radius length in the TFCC injured group was 4.83 mm less than the 7.19 mm in the uninjured group, and the DRUJ distance of 2.95 mm in the injured group was significantly greater than the 1.83 mm in the uninjured group.

**Table 3:** Comparing of the demographic data and radiologic parameter of the two groups

	Uninjured group	Injured group	*p* value
Total	79	93	
Sex (male/female)	41:47	35:49	0.338
Age	57.5	55.6	0.540
Injured side (L:R)	36:44	47:45	0.478
Fracture type (intra/extra-articular)	59:20	80:13	0.074
Radiologic parameter			
**Radius length (mm)**	**7.19**	**4.83**	**0.044**
Radius inclination (°)	18.27	17.9	0.439
**DRUJ distance (mm)**	**1.83**	**2.95**	**0.040**
Palmer tilt angle (°)	0.69	− 9.54	0.092
Sagittal translation (mm)	− 0.66	− 1.43	0.183

Radius length and DRUJ distance had significant relevance with TFCC injury in distal radius fracture (Student’s t test, *p* < 0.05)

*L* Left; *R* Right; *DRUJ* Distal radioulnar joint

While the radius length and DRUJ distance between the uninjured and injured TFCC patients were significantly related to the pattern of TFCC injury, a shorter radius length was related to type lB TFCC injury (Table 4).

**Table 4:** Comparing radius length and DRUJ distance of different types of TFCC injury

Radiologic parameter	TFCC injury (Palmer’s classification)				
	_1A_	_IB_	_1C_	_1D_	*p* value
Radius length (mm)	_6.44_	_3.73_	_4.88_	_5.81_	_0.041_
DRUJ distance (mm)	_2.54_	_3.06_	_2.61_	_3.33_	_0.398_

Radius length differed in different types of TFCC injury which showed shorter in type 1B (ANOVA, *p* < 0.05)

*TFCC* Triangular fibrocartilage complex; *DRUJ* Distal radioulnar joint

**Fig. 2:**
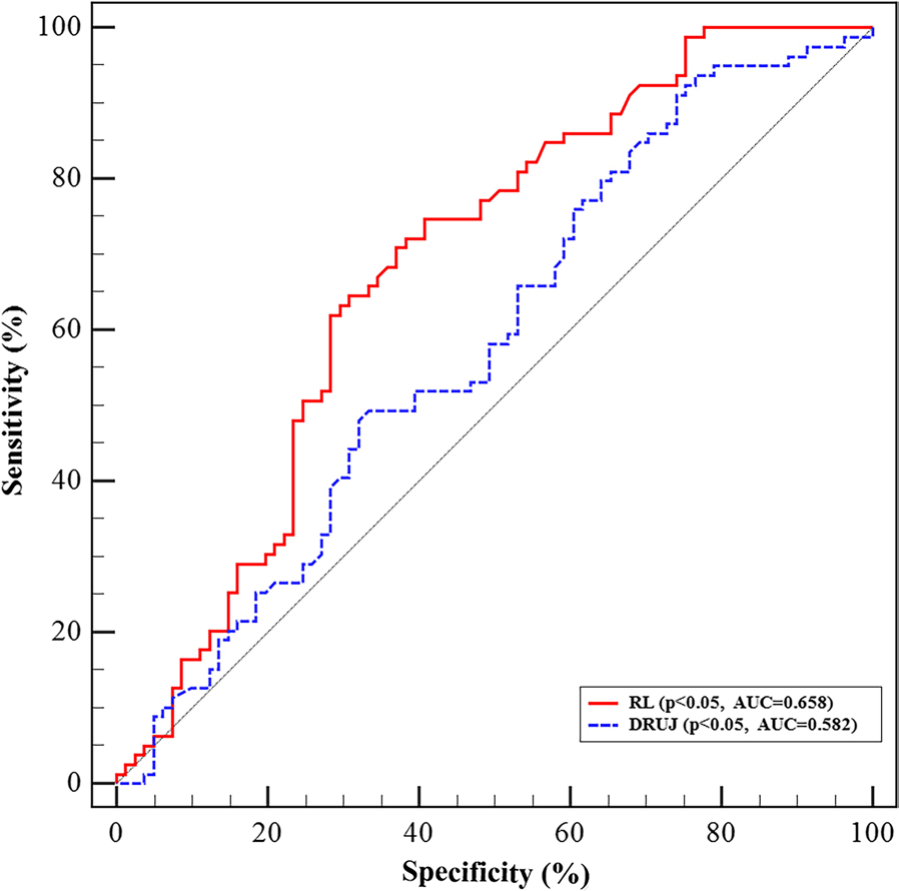
ROC curves of radius length and DRUJ distance for TFCC injury. The AUC value of RL was 0.658 and DRUJ was 0.582. RL, radius length; DRUJ, distal radioulnar joint

The analysis of the receiver operating characteristic curve revealed a statistical correlation between radius length and DRUJ distance and traumatic TFCC injury in distal radius fracture (Fig. 2); notably, the ROC curve of radius length presents AUC value slightly higher than DRUJ distance. However, both of them showed no significant sensitivity and specificity, and perhaps they could be the basis for determining whether TFCC is an injury.

## Discussion

TFCC injury is a frequent soft tissue injury that has been observed to accompany distal radius fractures, which are rather common. The Palmer’s classification method states that TFCC injury of Palmer type IB is known to lead to instability of the DRUJ [17, 18]. In addition, instability of the DRUJ can lead to early onset of arthritis, decreased grip strength, a restriction of the range of motion (ROM) of the wrist joint, and persistent discomfort in the wrist if it is not properly treated. It is also recognized as a factor with a poor outcome for distal radius fractures [19]. Distal radius radiographs are utilized for diagnosis, treatment planning, fracture reduction evaluation, and healing monitoring. If these radiographic characteristics could help us determine whether a fracture has occurred while also initially determining whether the TFCC is injured or not, that would be very significant.

Our investigation showed that traumatic TFCC injury occurred in 54.1% of patients with DRF (75.6% in C3 type). 1B TFCC injury was most common, with an incidence of 26.7% in all patients with DRF and 49.5% (table not shown) in the TFCC injury group. Moreover, the general radius length and DRUJ distance of the TFCC injury group were 4.83 mm and 2.95 mm, respectively. A shorter radius length was related to lB TFCC injury. Radius length and DRUJ distance cannot be utilized as predictors even if they have a statistical correlation with TFCC injury.

Studies are conflicting regarding the relationship between traumatic TFCC injury and radiologic parameters in patients with DRF. K. Kasapinova et al. [20] noted that the initial radiograph of a distal radius fracture does not predict a triangular fibrocartilage complex injury. In contrast, Beom-Soo et al. [21] mentioned that the shortening of the distal radius, causing peripheral soft tissue of the ulnar side to become tauter, is highly relevant with regard to the pattern of TFCC injury. However, in those studies, relatively little clinical data were collected. This study has the most numerous strengths in that it collected complete imaging data, including X-ray, CT, and MRI from 172 patients, and identified the relationship between radius length as well as DRUJ distance and TFCC injury through imaging modalities. Moreover, this study demonstrates that type 1B TFCC injuries are more common in distal radius fractures and that TFCC injuries are not necessarily the worst in intra-articular fractures; for example, the incidence of TFCC injury is higher in type B3 distal radius fractures than in C1 and C2 fractures.

Distal radius fracture displacing more than 4 mm and tilt radially beyond 0° and dorsally > 10° have significant clinical implications because distal radius fractures with shortening or dorsal tilt beyond the above values will influence DRUJ instability. As early as 1997, Richards et al. [9] found a relationship between the initial displacement of DRF and the presence of TFCC injury which was associated with significantly greater shortening and dorsal angulation of the radius compared with fractures where the TFCC was uninjured. Kwon et al. [22] reported that initial distal radial shortening of more than 6.0 mm is one of the risk factors for TFCC injury and DRUJ instability. Previous studies have shown that an increase in DRUJ distance is an independent risk factor for DRUJ instability in distal radius fractures [7]. In addition, Omokawa et al. [23] reported that the DRUJ gap distance is the most important predictor of TFCC injury accompanying an unstable distal radius fracture. In this study, patients with TFCC injury tended to have a shortened radius and widened DRUJ distance, although they had no predictive value for TFCC injury in DRF. Prior research has shown a correlation between TFCC injury and the amount of dorsal or volar angulation in the fracture [24, 25]; however, there was no significant relationship between the palmer tilt angle and other radiologic parameters and TFCC injury in this study.

Since 1B TFCC injury may result in DRUJ instability, some researchers advised treating the TFCC injury if DRUJ instability is detected after the fixation of DRF [26]. However, DRUJ instability may not be reliably detected by manual stress tests, such as the radioulnar stress test [27, 28]. Perioperative manual stress testing, in particular, cannot effectively detect DRUJ instability because edema or subcutaneous hematoma around the wrists would make identification difficult. According to our research, a shorter radius length was associated with 1B TFCC injury and could serve as a significant reference value. Specifically, radial length of less than 5 mm following DRF was associated with a higher risk of 1B TFCC injury; however, a slight variation in radius length does not always mean the absence of TFCC injury. Although the significant instability of the DRUJ due to 1B TFCC injury would be the cause of the residual ulnar wrist pain, which type of 1B TFCC injury arouses the DRUJ instability which results in the residual symptoms are still unknown and further investigation should be needed.

The treatment goals for DRF are painless and fully functional wrist motion. Although the treatment of DRF with VLP fixation reportedly achieves satisfactory outcomes, several researchers have reported that some patients experience ulnar wrist pain after surgical treatment of DRF [29]. TFCC injuries are a recognized cause of ulnar side wrist pain, resulting in unsatisfactory outcomes [5, 6]. Many authors have recommended surgical repair of the TFCC in distal radius fractures to prevent the adverse effect of instability of the DRUJ and ulnar side wrist pain [30]. In contrast, authors who recommend conservative treatment have reported that long-arm splinting for 1 month after internal fixation of the distal radius fracture with instability of the DRUJ could result in a satisfactory outcome [31]. Moreover, Jemin et al. [32] found that there were no statistically significant differences in the clinical outcomes between surgical and conservative treatment. Although controversial, we must treat TFCC injury with the proper treatment methods to minimize their negative effects on the patient as much as possible. In clinical practice, it is possible to select a customized treatment plan following appropriate patient discussion. Even though the majority of patients choose a conservative course of therapy, the surgeon should still be well-informed on the current debates surrounding the management of TFCC injury as well as the potential postoperative consequences. The height of the radius should be restored as much as possible during surgery in cases with DRF with substantial shortening of the radius (particularly if the radius length is less than 5 mm). Furthermore, it is recommended to use a wrist brace or splinting for 4 to 6 weeks to fix the wrist in order to prevent TFCC injury and potential DRUJ instability. Still, further investigation is required as this is only the researchers’ observation.

There are a few limitations to this study. The main limitation is in the measurement and categorization of the relevant image data. This study involves a large number of radiologic parameters, and measurement on X-ray is prone to bias, but it is truly the most straightforward and constrained approach available. Measurement by CT may be more accurate, but there are many problems in practice, such as the measurement of angles. The classification of TFCC injury by analyzing MRI is also subjective, and perhaps the results would be more accurate afterward with arthroscopy. Another limitation is that this study focused on radiologic parameters and ignored the influence of other factors on TFCC injury, such as ulnar styloid fracture, which may have some relevance to TFCC injury [33, 34]. Therefore, further investigation of the differences in the clinical outcomes of distal radius fractures is needed.

## Conclusion

The present study has shown that 1B TFCC injury is most common in patients with DRF and concomitant TFCC injury. In addition, both radius length and DRUJ distance have a statistical correlation with TFCC injury and patients with TFCC injury tend to have a shortened radius and wider DRUJ distance, although they have no predictive value for TFCC injury in DRF.

## Data Availability

All the data in this study came from the First People’s Hospital of Changzhou, and all the data were recorded through the contemporaneous preservation of documents.
